# Notes on Some Experiments, Illustrating the Influence of the Vis Medicatrix, and of the Imagination, in the Cure of Diseases

**Published:** 1847-01

**Authors:** 


### II. NOTES OF SOME EXPERIMENTS, ILLUSTRATING THE INFLUENCE OF THE VIS MEDICATRIX, AND OF THE IMAGINATION, IN THE CURE OF DISEASES.

BY A NAVAL SURGEON.*

(*In a Letter to John Forbes, M.D., F.R.S.*)

[In our anxious desire to rouse the attention of the profession to a philosophical investigation of the real powers and actions of medicines, we willingly give a place in our pages to the present communication. Its bearing at once on the nonentities of homœopathy and the too strong realities of heroic medication, and also on the nature of the logic too current in the profession—is sufficiently obvious. We should think it would be equally obvious to every one who had taken the pains to read all our preceding papers on this subject, that, in giving publicity to such a document as this, we do *not* in any way advocate the propriety of leaving diseases to Nature, or of withholding any of the rational aids of ordinary medicine; much less, that we hold out to our readers our correspondent's plan of working with imaginary remedies, as one to be followed in ordinary practice. We here merely record the repetition, in an authentic form, of an old experiment, which we deem of some importance at the present time, and which the author meant simply as an experiment. Perhaps we should have taken this opportunity of offering, in our own name, some comments on certain misapprehensions of our views respecting the treatment of diseases recently advocated in this Journal, had not this been so admirably done for us by Dr. Combe, in the communication immediately preceding this. To this communication we request the best attention of our readers.

We must also say one word in reference to the cases of fever noticed in the present paper. Of course the number of instances given is infinitely too small to authorize any *general* inferences respecting treatment. The cases are, however, valuable *as instances*, as far as they go: *valeant quantum*.]

H.M.S. ——— Piræus of Athens, October 2d, 1846.

My dear Sir,—The correspondence, published in the July Number of your Journal, descanting on and highly approving of your invaluable article entitled Homœopathy, Allopathy, &c., which appeared in your January Number, has only just now met my eye. This correspondence has recalled to my mind the intention I had previously entertained, of offering to you my humble tribute of thanks, for having given to the world a paper the effect of which is likely to be attended with the most beneficial results, by promulgating doctrines and opinions of the highest importance to medicine; the truth of which has (at least partially) been long felt, although few have had the inclination, and none the courage, publicly to avow it. I now proceed to accomplish what I only before had purposed.

For a long period I have been getting more and more sceptical as to the curative effects of certain medicines in many diseases; while the use of the lancet, and other violent depressing means, have been almost totally omitted in my practice for nearly ten years. While on the coast of Africa, and in medical charge of the Island of Ascension, about ten years since, I fancied I saw great reason to alter the violent depleting mode of treatment then in vogue in those countries.

* The writer of this communication is an officer of long standing and much experience. His name and high character are known to the Editor.

In 1838, a bad attack of marsh fever broke out in the ship I was serving in, in the Mediterranean. My assistant was one of the sufferers. I was, consequently, left entirely to act on my own responsibility. Being in the greatest doubt as to what line of treatment was likely to be attended with the greatest amount of temporary alleviation of the distressing symptoms, as well as what was to tend most to the security of my patients against any permanent bad effects from the epidemic, I determined on running some risk with the first few, in the hope of being the better able to relieve the sufferings of those who came after. Acting upon this determination, I took the first twelve cases, officers and men, as they presented themselves. The fever in all these having assumed the ardent form, blood was abstracted from four, in quantities which, at the time, were considered sparing, as all were bled in the erect position, and from a free opening, and in two of these four the bleeding was repeated during the evening of the first day; laxatives were given, and the system attempted to be kept under the influence of antimony. Two were treated according to the mode then just made public, by M. Malliot—viz. by large doses of quinine from the very commencement. Two, after having had an emetic and purgative dose, were put under active mercurial treatment. Two had each a dose composed of the following ingredients: T. digitalis, T. opii, Vin. antimonialis, Vin. ipecacuanhæ, of each ʒiss. This dose was repeated after six hours; and after the profuse perspiration brought out by these draughts had in part subsided, the patients were simply watched, to see that they had effervescing or other palatable drinks in what quantity they chose. Two were put to bed, their skins freely sponged with vinegar, and watery drink given ad libitum, and so left, without even a dose of laxative medicine. Now for the result. Of the first four (the parties who were bled), two died, and one, subsequently, required to be invalided and sent home. Of the two who had ℈j doses of quinine, one died, and one subsequently required to be invalided. Of the two who were treated by mercury one died, and one had a most protracted convalescence, and died some two years after, without having ever fully regained his strength. The whole of the others were, after longer or shorter periods of convalescence, enabled to return to their duties, having, to all appearance, regained their original vigour. The fever continued in the ship until we were enabled to change our locality—viz. from the Gulf of Scanderoon (situated between Asia Minor and Syria) to the mouth of the Dardanelles. But as the whole of the remaining cases were treated in compliance with some special indication, I am inclined to consider the results as of less value. Need I add that since that period the patient labouring under fever, who has been solely under my charge, has never been bled, or that but very little medicine, in the treatment of either Mediterranean or West Indian fever, has been expended in my practice? And yet my returns, so far as I am aware, show no more fatal results than those of others similarly situated, but whose mode of treatment is much more active. So much for letting Nature have her way in those diseases which appear to arise from the absorption of specific poisons, as in the case of marsh and other fevers.

I am almost as sceptical as to the necessity of large bleedings, in inflammation of the contents of the larger cavities, as I am in fever. Convinced, in my own mind, that I had seen diseases of the chest more especially, not only kept up, but hurried on to a fatal termination by the large and repeated bleedings practised for their removal, I long ago began to be chary of following such practice. And here again I may mention that for years I have only once used a lancet, in the treatment of such inflammatory cases as have come under my care; and in that one (a case of inflammation of the bowels) I yielded with reluctance to the solicitations of my assistant, not from any impression of its absolute necessity. The man got well; but so do other men who sustain no such loss. It must not, however, be imagined that I mean to convey the belief that I am prepared to doubt the efficacy of all curative means, or that I should be able to stand by and see active inflammation take its course, without making an effort to stop its progress. Very far from this. I only hold that in many cases where, in ordinary practice, the lancet is again and again had recourse to, the previous use of digitalis and antimony, prussic acid, opium, &c., will lead to results of a more satisfactory nature, than when the depleting system is practised to a large extent.

Since the year 1837, I have been in the habit of occasionally treating some few of my patients, whom I could neither dose nor reason into good health, by a different method still. And this method will, perhaps, be best illustrated by one or two examples.

Case I. A very intelligent officer had suffered for some years from violent attacks of cramp in the stomach. He had tried almost all the remedies usually recommended for the relief of this distressing affection; and for a short period prior to coming under my care, the trisnitrate of bismuth had been attended with the best results. The attacks came on about once in three weeks, or from that to a month, unless when any unusual exposure brought them on more frequently. As bismuth had been so useful, it, of course, was continued; but notwithstanding that it was increased to the largest dose that its poisonous qualities would justify, it soon lost its effect. Sedatives were again applied to; but the relief afforded by these was only partial, while their effect on the general system was evidently very prejudicial. On one occasion, while greatly suffering from the effect of some preparation of opium, given for the relief of these spasms, he was told that on his next attack he would be put under a medicine which was generally believed to be most effective, but which was rarely used, in consequence of its dangerous qualities; but that, notwithstanding these, it should be tried, provided he gave his consent. This he did willingly. Accordingly, on the first attack after this, a powder, containing four grains of *ground biscuit*, was administered every seven minutes, while the greatest anxiety was expressed (within the hearing of the party), lest too much should be given. The fourth dose caused an entire cessation of pain. Half-drachm doses of bismuth had never procured the same relief in less than three hours. For four successive times did the same kind of attack recur, and four times was it met by the same remedy, and with like success! After this my patient was ordered to join another ship, on a different station.

Case II. A seaman had suffered from four successive attacks of constipation. So far as could be detected, there was no organic disease to account for its occurrence. The symptoms were such as usually follow protracted constipation of the bowels; and on all four occasions large and repeated doses of the strongest purgatives (croton oil included), powerful enemata, cold affusion, and hot baths, had all been required to be persevered in to procure relief. On the fifth attack he was put under grs. ij of bread-pill every seven minutes; much anxiety being, of course, expressed to guard against any over-dose, as well as to watch the effect of what was thus given. Within two hours he became sick (*one of the symptoms expected from the medicine*); and his bowels were freely open almost immediately after; nor did they again become constipated, so far as I am aware.

Case III. In July, 1845, the company of H. M. S. ——— were attacked with an epidemic bowel complaint, terminating in simple diarrhea in some, but going on to dysentery in many. In every one of the latter cases tapeworms (whether a cause, or merely an effect, I am unable as yet to divine) showed themselves. Amongst others who suffered was H. B., a first-class petty officer, who had but a mild attack of dysentery, but who was much distressed towards the latter part of his attack by tapeworm appearing in considerable quantities. As the dysenteric symptoms disappeared, these worms were attempted to be dislodged by every means that could be devised, and for a time it was supposed these means had been successful; but, as I feared, at too great a sacrifice, seeing that the pain arising (as I fancied) from the large doses of powerful medicine necessary to effect this difficult object, continued around the pyloric orifice of the stomach and upper portion of the small intestines, to be most distressing. Counter-irritants were applied until the skin became callous, sedatives administered until the man's senses became muddled, but no course of treatment seemed to afford the least relief This being so, I determined to try the effect of mental influence. Stating to him, as I did to the other men, that as his disease was most obstinate, so was it necessary to have recourse to desperate means to relieve it; that, with his sanction, I would therefore put him under a medicine which it was most necessary to watch with the greatest attention, lest its effects should prove most prejudicial, perhaps fatal, &c. Having by these statements made an impression, it became necessary to keep it up. This was done by repeated visits at all hours of the day and night, and by expressing on these occasions the most intense anxiety as to the effect of the very powerful and dangerous medicament. This was not a case in which a sudden effect could be expected to be produced, whatever might be the means employed. Symptoms of disease existed which bore too close a resemblance to those of an organic order, to admit of hope of a sudden, if even of tardy relief. Hence the pills (bread, of course) were given every sixth hour only. Within twenty-four hours the man's sufferings were decidedly less. Within four days he was almost free from pain. On the sixth day he was quite so; his pills were omitted, and at the end of a fortnight he was again at duty, with a clear eye, a healthy skin, and was rapidly regaining his flesh. Here, as in most cases where this method has been tried, the diet and drink have been left unrestricted. Occasionally, however, it became necessary to taboo some article, lest its coming in contact with the remedy might prove most destructive; in other words, articles are occasionally forbidden when the mind seems to be inclined to lose sight of what must be made the all-important subject of thought by night and day. The wonderful improvement in this man's state was frequently commented on by both officers and men, who, of course, were, and still are, as little acquainted with the means employed as the patient himself was.

It may be said that this case, as here given, goes for nothing, in so far as it does not show that the pains were anything but casual; in which case, any other mode of treatment, or very likely no mode at all, would have been equally successful; or it may be again, as it has before been said, that it was altogether feigned, and that the commanding officer would have made a better and quicker cure. I think not; and for the following reasons: The man's flesh had wasted, his eye became sunken, his skin sickly in hue, as well as in feeling, his sleep, when he had any, was of the most disturbed order. But, more than all, the pain after some weeks returned, and the other bad symptoms followed in its wake; yet both it and they were again relieved a second time by the same means. While suffering from a third attack, he was sent to the Royal Naval Hospital at Malta, and where, after much suffering, he brought up by vomiting a portion of the mucous membrane of one of the small intestines, distinctly marked by two, at least, of the valvulæ conniventes. I am assured by one of the officers of that establishment, that he most carefully examined this ejected matter, and that its characters were so marked that there could be no room for a doubt as to what it was. This being so, we have pretty clear proof that disease existed long before this slough was thrown off; and that even this organic disease was suspended on two occasions by mental influence only.

It is clear that there are great and evident objections to this mode of treatment becoming generally useful. The fact of its being based and built up on deception renders it an unworthy means for an intelligent practitioner to have recourse to, even when his sole object is the relief of suffering. Nor is the fact that publicity would render it at once inutile, less certain of preventing it being followed. But though neither fit to be generally practised by others, or long followed by me, the truth which the above cases, and others like them, inculcate, ought to be made known; the more especially so at a time when the homœopathist is expending his powers in showing to the world how beautifully his infinitesimal doses act and react upon disease; as it may tend to convince some that if in many cases they would leave their doses, whether large or small, altogether alone, they might, perhaps, be equally near their purpose; or I ought rather to say, that their patients might, perhaps, be equally near a sound state of health.

I am, my dear Sir,
Yours faithfully,
* * * *

